# A novel algorithm for finding top-k weighted overlapping densest connected subgraphs in dual networks

**DOI:** 10.1007/s41109-021-00381-8

**Published:** 2021-06-05

**Authors:** Riccardo Dondi, Mohammad Mehdi Hosseinzadeh, Pietro H. Guzzi

**Affiliations:** 1 Department of Science, University of Bergamo, Bergamo, Italy; 2 Department of Surgical and Medical Sciences, Magna Graecia University, Catanzaro, Italy

**Keywords:** Dual networks, Network mining, Dense subgraphs, Graph algorithms

## Abstract

The use of networks for modelling and analysing relations among data is currently growing. Recently, the use of a single networks for capturing all the aspects of some complex scenarios has shown some limitations. Consequently, it has been proposed to use Dual Networks (DN), a pair of related networks, to analyse complex systems. The two graphs in a DN have the same set of vertices and different edge sets. Common subgraphs among these networks may convey some insights about the modelled scenarios. For instance, the detection of the Top-k Densest Connected subgraphs, i.e. a set k subgraphs having the largest density in the conceptual network which are also connected in the physical network, may reveal set of highly related nodes. After proposing a formalisation of the approach, we propose a heuristic to find a solution, since the problem is computationally hard. A set of experiments on synthetic and real networks is also presented to support our approach.

## Introduction

In last years, the use of networks to manage and analyse experimental data in many fields has grown (Cannataro et al. 2010; Barabási 2011). For instance, in computational biology associations among biological molecules (such as genes, proteins, small lipids etc.), are usually modelled as graphs. Data collected from social networks are modelled using graph theory and their analysis may shed light into association patterns among users (Sapountzi and Psannis 2018; Abatangelo et al. 2009; Clark and Kalita 2014; Faisal et al. 2015; Cannataro et al. 2010)

Usually, data are modelled using a single network whose nodes represent entities and edges their relations. Then, the topological analysis of the networks, i.e. global or local structures (Cannataro et al. 2010), finds context specific properties such as groups of related genes in biology or users in social networks (Liu et al. 2018). More recently, some works demonstrated that the use of a single network may not be able to capture all the relationships among elements considered, therefore some complex models have been introduced such as heterogeneous networks (Milano et al. 2020) or dual networks (Wu et al. 2016). A dual network is a pair of related graphs sharing the same node set, with two different edge sets. One network has unweighted edges, and it is called *physical graph*. The second one has weighted edges and it is called *conceptual graph*. For example, in biology dual networks have been used to model interactions among genetic variants (Phillips 2008), where genetic interactions are modelled using the physical network and the quantitative effects of these interactions are modelled with the conceptual one.

An interesting problem in dual networks is the Densest Connected Subgraph (DCS) problem, that is finding a common subgraph between the two networks that has two properties: it is connected on the physical one and it is densest in the conceptual one. A DCS in a dual network may convey relevant information. For instance (Guzzi et al. 2020), showed that DCS may suggest missing links in social networks, capture similar interests among authors in a co-authorships dual network, where physical network represents co-authors and the conceptual network is used to model topics shared.

The relevance of problem arises in many real life scenarios. For instance in Phillips (2008) authors extracted a DCS from dual networks to analyse interactions between genetic variants and their strength. Given two input graphs $G_{c}(V,E_{c})$ (undirected and edge-weighted), and $G_{p}(V,E_{p})$ (undirected and unweighted), the problem consists in finding a subset of nodes $I_{s}$ that induces a densest community in $G_{c}$ and a connected subgraph in $G_{p}$. As proved in Wu et al. (2016), the DCS problem is NP-hard, since it may be reduced from the Set Cover problem (Karp 2009). Therefore there is the need for novel heuristics and computational approaches to solve it. Here we focus on a generalisation of this problem, since we search for a set of (overlapping) common subgraphs, that are connected in the physical network and densest in the conceptual network, i.e. top-k weighted overlapping densest connected subgraphs. The identification of top k-densest overlapping subgraphs in a network has been considered in Galbrun et al. (2016); Dondi et al. (2019); Hosseinzadeh (2020).

**Fig. 1:**
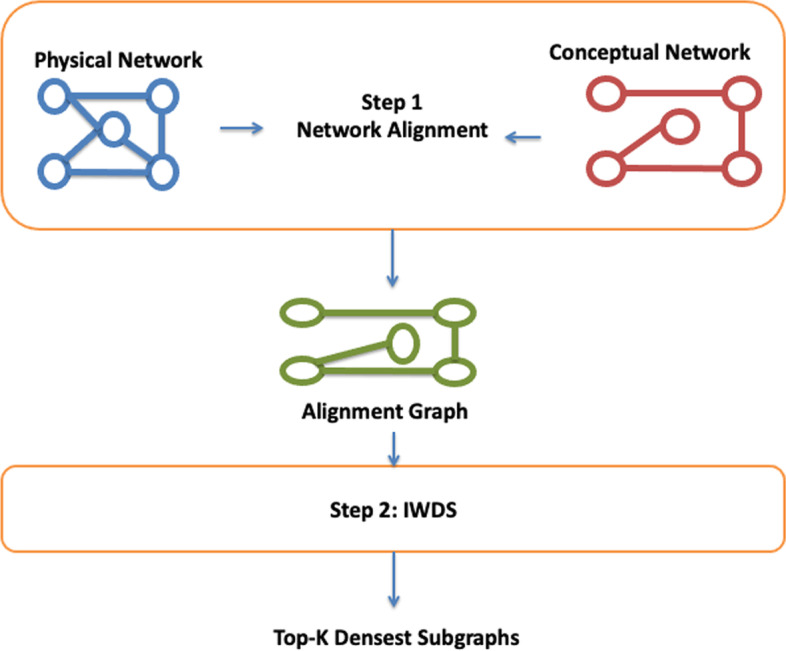
Workflow of the proposed approach. In the first step the input conceptual and physical networks are merged together using a network alignment approach; then Weighted-Top-k-Overlapping DCS is applied on the alignment graph. Each extracted subgraph induces a connected subgraph in the physical network and one of the top-k overlapping weighted densest subgraph in the conceptual one

Our approach is based on a two step strategy: first a single alignment graph is built from the dual networks Guzzi and Milenković (2017); Milano et al. (2020), then we look for dense subgraphs in this network with an ad-hoc heuristic. Notice that these subgraphs correspond to dense subgraphs in the conceptual networks and connected subgraphs in the physical one, therefore they are solutions of the initial problem. Figure 1 depicts the workflow of our approach.

Considering the state of the art, we should note that we allow more flexibility with respect to other works such as Wu et al. (2016). In this work authors do not consider overlapping subgraphs and their approach is limited to the exact correspondence of nodes between networks. On the other hand, with respect to other approaches for finding densest subgraphs in a network (Balalau 2015; Galbrun et al. 2016; Dondi et al. 2019; Guzzi and Cannataro 2010), we consider weighted networks, an extension that can be useful in many contexts, in particular for biological and social networks.

We provide an implementation of our heuristic, and we show the effectiveness of our approach on synthetic datasets and on four real networks (a social network, two biological networks, a co-authorship network). The experimental results confirm the effectiveness of our approach.

The paper is structured as follows: “Related work” section discusses related works, “Definitions” section gives definitions and formally introduces the problem we are interested into. “The proposed algorithm” section presents our heuristic; “Experiments” section discusses the case studies; finally “Conclusion” section concludes the paper.

## Related work

Many complex systems cannot be efficiently modelled using a single network without losses of information. Therefore the use of dual networks is growing (Wu et al. 2016; Sun and Kardia 2010). These applications span a large number of fields as introduced before: from bioinformatics to social networks. In genetics, dual networks are used to describe and analyse interactions among genetic variants. They can discover the common effects among multiple genetic variants (Sun and Kardia 2010), using a protein–protein interaction network that represents physical interactions and a weighted network that represents the relations between two genetic variants, usually measured by statistical tests.

A relevant problem in network analysis is that of discovering dense communities, as they represent strongly related nodes. The problem of finding communities in a network or a dual network is based on the specific model of dense or cohesive graph considered. Several models of cohesive subgraph have been considered in the literature and applied in different contexts. One of the first definition of a cohesive subgraph is a fully connected subgraph, i.e. a clique. However, the determination of a clique of the maximum size, also referred to as the Maximum Clique Problem, is NP-hard (Hastad 1996), and it is difficult to approximate (Zuckerman 2006). Moreover, in real networks communities may have missing edges; therefore, the clique model is often too strict and may fail to find some important subgraphs. Consequently, many alternative definitions of cohesive subgraphs that are not fully interconnected have been introduced, including *s*-club, *s*-plex and densest subgraph (Komusiewicz 2016; Dondi et al. 2019).

A *densest subgraph* is a subgraph with maximum density (where the density is the ratio between the number of edges and number of nodes of the subgraph) and the Densest-Subgraph problem asks for a subgraph of maximum density in a given graph. The problem can be solved in polynomial time (Goldberg 1984; Kawase and Miyauchi 2018) and approximated within factor $\frac{1}{2}$ (Asahiro et al. 2000; Charikar 2000). Notice that the Densest-Subgraph problem can be extended also to edge-weighted networks.

Recently, Wu et al. (2016), proposed an algorithm for finding a densest connected subgraph in a dual network. The approach is based on a two-step strategy. In the first step, the algorithm prunes the dual network without eliminating the optimal solution. In the second step, two greedy approaches are developed to build a search strategy for finding a densest connected subgraph. Briefly, the first step finds the densest subgraph in the conceptual network. The second step refines this subgraph to guarantee that it is connected in the physical network.

In this contribution we use an approach based on local network alignment (LNA) that aims to find (relatively) small regions of similarity among two or more input networks. Such regions may be overlapping or not, and they represent conserved topological among networks. For instance, in protein interaction networks these regions are related to conserved motifs or pattern of interactions (Guzzi and Milenković 2017). LNA algorithms are usually based on building an intermediate structure, defined as alignment graph, and on the subsequent mining of it (Milano et al. 2020). For instance, Ciriello et al. (2012) and its successor AlignMCL (Mina and Guzzi 2014) are based on the construction of alignment graphs (see related papers for complete details about the construction of the alignment graph). GLAlign (Global Local Aligner), is a new local network alignment methodology (Milano et al. 2018) that mixes topology information from global alignment and biological information according to a linear combination schema, while the more recent L-HetNetAligner (Milano et al. 2020) extends the local alignment to heterogeneous networks.

While the literature of network mining has mainly focused on the problem of finding a single subgraph, recently the interest in finding more than a subgraph has emerged (Balalau 2015; Galbrun et al. 2016; Dondi et al. 2019; Hosseinzadeh 2020; Cho et al. 2013). The proposed approaches usually allows overlapping between the computed dense subgraphs. Indeed, there can be nodes that are shared between interesting dense subgraphs, for example hubs. The proposed approaches differ in the way they deal with overlapping. The problem defined in Balalau (2015) controls the overlap by limiting the Jaccard coefficient between each pair of subgraphs of the solution. The Top-k-Overlapping problem, introduced in Galbrun et al. (2016), includes a distance function in the the objective function. In this paper, we follow this last approach and we extend it to weighted networks.

## Definitions

This section introduces the main concepts related to our problem.

### Definition 1

Dual Network.

A Dual Network (DN) $G(V,E_c,E_p)$ is a pair of networks: a conceptual weighted network $G_c(V,E_c)$ and a physical unweighted one $G_p(V,E_p)$.

Now, we introduce the definition of weighted density of a graph.

### Definition 2

Density.

Given a weighted graph *G*(*V*, *E*, *weight*), let $v \in V$ be a node of *G*, and let

$$\begin{aligned}vol(v)=\sum _{w:(v,w)\in E}weight(v,w)\end{aligned}$$

be the sum of the weights of the edges incident in *v*. The density of the weighted graph *G* is defined as

$$\begin{aligned} \rho (G)=\frac{\sum _{v \in V}vol(v)}{|V|}. \end{aligned}$$

Given a graph (weighted or unweighted) *G* with a set *V* of nodes and a subset $Z \subseteq V$, we denote by *G*[*Z*] the subgraph of *G* induced by *Z*. Given $E' \subseteq E$, we denote by $weight(E')$ the sum of weights of edges in $E'$. Given a dual network we denote by $G_{p}[I]$, $G_{c}[I]$, respectively, the subgraphs induced in the physical and conceptual network, respectively, by the set $I \subseteq V$.

A densest common subgraph *DCS*, formally defined in the following, is a subset of nodes *I* that induces a connected subgraph in the conceptual network and a connected subgraph in the physical network.

### Definition 3

Densest Common Subgraph.

Given a dual network $G(V,E_c,E_p)$, a densest common subgraph in $G(V,E_c,E_p)$ is a subset of nodes $I \subseteq V$ such that $G_p[I]$ is connected and the density of $G_c[I]$ is maximum.

In this paper, we are interested in finding $k \ge 1$ densest connected subgraphs. However, to avoid taking the same copy of a subgraph or subgraphs that are very similar, we consider the following distance functions introduced in Galbrun et al. (2016).

### Definition 4

Let $G(V,E_c,E_p)$ be a dual network and let *G*[*A*], *G*[*B*], with $A, B \subseteq V$, be two induced subgraphs of *G*. The distance between *G*[*A*] and *G*[*B*], denoted by $d: 2^{V} \times 2^{V} \rightarrow \mathbb {R_{+}}$ has value equal $2-\frac{|A \cap B|^2}{|A||B|}$ if $A \ne B$, else is equal to 0.

Notice that $2-\frac{|A \cap B|^2}{|A||B|}$ decreases as the overlapping between *A* and *B* increases.

Now, we are able to introduce the problem we are interested into.

### Problem 1

Weighted-Top-k-Overlapping DCS

**Input:** A dual network $G(V,E_c,E_p)$, a parameter $\lambda > 0$.

**Output:** a set ${\mathcal {X}} = \{ G[X_1], \ldots , G[X_k] \}$ of *k* connected subgraphs of *G*, with $k \ge 1$, such that the following objective function is maximised:

$$\begin{aligned} \sum _{i=1}^{k} \rho (G_c[X_i])+ \lambda \sum _{i=1}^{k-1} \sum _{j=i+1}^k d(G[X_i],G[X_j]) \end{aligned}$$

Weighted-Top-k-Overlapping DCS, for $k \ge 3$, is NP-hard, as it is NP-hard already on an unweighted graphs (Dondi et al. 2019). Notice that for $k=1$, then Weighted-Top-k-Overlapping DCS is exactly the problem of finding a single weighted densest connected subgraph, hence it can be solved in polynomial time (Goldberg 1984).

### Greedy algorithms for DCS

One of the ingredient of our method is a variant of a greedy algorithm for DCS, denoted by Greedy, which is an approximation algorithm for the problem of computing a connected densest subgraph of a given graph. Given a weighted graph *G*, Greedy (Asahiro et al. 2000; Charikar 2000) iteratively removes from *G* a vertex *v* having lowest *vol*(*v*) and stops when all the vertices of the graph have been removed. It follows that at each iteration *i*, with $1 \le i \le |V|$, Greedy computes a subgraph $G_i$ of *G*. The output of this algorithm is a densest of subgraphs $G_1, \ldots , G_{|V|}$. The algorithm has a time complexity $O(|E| + |V| \log |V|)$ on weighted graphs and achieves an approximation factor of $\frac{1}{2}$ (Asahiro et al. 2000; Charikar 2000).

We introduce here a variant of the Greedy algorithm, called V-Greedy. Given an input weighted graph *G*, V-Greedy, similarly to Greedy, at each iteration *i*, with $1 \le i \le |V|$, removes a vertex *v* having lowest *vol*(*v*) and computes a subgraph $G_i$, with $1 \le i \le |V|$. Then, among subgraphs $G_1, \ldots , G_{|V|}$, V-Greedy returns a subgraph $G_i$ that maximises the value:

$$\begin{aligned} \rho (G_i) + 2\left( \frac{\rho (G_i)}{|V_i|}\right) . \end{aligned}$$

Essentially, when selecting the subgraph to return among $G_1, \ldots , G_{|V|}$, we add to the density the correction factor $2(\frac{\rho (G_i)}{|V_i|})$. This factor is added to avoid returning a subgraph that is not well-connected in terms of edge connectivity, that is it contains a small cut. For example, consider a graph with two equal size cliques $K_1$ and $K_2$ having the same (large) weighted density and a single edge of large weight connecting them. Then the union of $K_1$ and $K_2$ is denser than both $K_1$ and $K_2$, hence Greedy returns the union of $K_1$ and $K_2$. This may prevent us to find $K_1$, $K_2$ as a solution of Weighted-Top-k-Overlapping DCS. In this example, when the density of $K_1$ and $K_2$ is close enough to the density of their union, V-Greedy will return one of $K_1$, $K_2$.

## The proposed algorithm

In this section we present our heuristic for Weighted-Top-k-Overlapping DCS in dual networks. The approach is based on two main steps:

1. First, the input networks are integrated into a single weighted alignment graph preserving the connectivity properties of the physical network
2. Second, the obtained alignment graph is mined by using an ad-hoc heuristic for Weighted-Top-k-Overlapping DCS based on the V-Greedy algorithm

### Building of the alignment graph

In the first step the algorithm receives in input: a weighted graph $G_c(V,E_c)$ (the conceptual graph); an unweighted graph $G_p(V,E_p)$ (the physical graph); an initial set (*seed nodes*) of node pairs *P*, where each pair defines a correspondence between a node of $G_c$ and a node of $G_p$; a distance threshold $\delta$ that represents the maximum threshold distance that two nodes may have in the physical network. For example, when $\delta$ is set to one, only adjacent nodes in both networks are considered.

Given the input data, the algorithm starts by building the nodes of the alignment graph. The alignment graph contains a node for each pair in *P*. The edges and weights of the alignment graph are defined as follows:

- An edge $\{u,v\}$ is defined in the alignment graph when the nodes corresponding to *u* and *v* are adjacent in $G_p$ and in $G_c$; the weight of $\{u,v\}$ is equal to the weight of the edge connecting the nodes corresponding to *u* and *v* in $G_c$
- An edge $\{u,v\}$ is defined in the alignment graph when *u* and *v* are adjacent in $G_p$ and have distance lower than $\delta$ in $G_c$; the weight of $\{u,v\}$ is equal to the average of the weights on a shortest path connecting the nodes corresponding to *u* and *v* in $G_c$.

### A heuristic for Weighted-top-k-overlapping DCS

In the second phase of our algorithm, we solve Weighted-Top-k-Overlapping DCS on the alignment graph *G* computed in phase 1 via a heuristic. We present here our heuristic for Weighted-Top-k-Overlapping DCS, called Iterative Weighted Dense Subgraphs (IWDS).

The heuristic starts with a set ${\mathcal {X}}= \emptyset$ and consists of *k* iterations. At each iteration *i*, with $1 \le i \le k$, given a set ${\mathcal {X}}= \{G[X_1],\ldots ,G[X_{i-1}]\}$ of subgraphs of *G*, IWDS computes a subgraph $G[X_i]$ and adds it to ${\mathcal {X}}$.

The first iteration of IWDS applies the V-Greedy algorithm (see “Greedy algorithms for DCS” section) on *G* and computes $G[X_1]$. In iteration *i*, with $2 \le i \le k$, IWDS applies one of the two following cases, depending on a parameter *f*, $0 < f \le 1$, and on the size of the set $C_{i-1} = \bigcup _{j=1}^{i-1} X_j$ (the set of nodes already covered by the subgraphs in ${\mathcal {X}}$).

*Case 1.* If $|C_{i-1}| \le f |V|$ (that is at most *f*|*V*| nodes of *G* are covered by the subgraphs in ${\mathcal {X}}$), IWDS applies the V-Greedy algorithm on a subgraph $G'$ pf *G* obtained by retaining $\alpha$ nodes ($\alpha$ is a parameter) of $C_{i-1}$ having highest weighted degree in *G* and removing the other nodes of $C_{i-1}$. $G'[X_i]$ is a weighted connected dense subgraph in $G'$, distinct from those in ${\mathcal {X}}$.

*Case 2.* If $|C_{i-1}| > f |V|$ (more than *f*|*V*| nodes of *G* are covered by the subgraphs in ${\mathcal {X}}$), IWDS applies the V-Greedy algorithm on a subgraph $G''$ of *G* obtained by removing $(1-\alpha )$ nodes (recall that $\alpha$ is a parameter of IWDS) of $C_{i-1}$ having lowest weighted degree in *G*. IWDS computes $G''[X_i]$ as a weighted connected dense subgraph in $G'$, distinct from those in ${\mathcal {X}}$.

*Complexity evaluation.*

We denote by *n* (by *m*, respectively) the number of nodes (of edges, respectively) of the dual network. The first step requires the analysis of both the physical and the conceptual graph, and the construction of the novel alignment graph. This requires ${\mathcal {O}}(n^2)(\hbox {calculation-edge-weights})$ time. The calculation of edge weights requires the calculation of a shortest path among all the node pairs in the physical graph using the Chan implementation (Chan 2012), therefore it requires ${\mathcal {O}}(n m_p)$ time ($m_p$ is the number of edges of the physical graph).

As for Step 2, IWDS makes *k* iterations. Each iteration applies V-Greedy on *G* and requires $O(m n \log n)$ time, as the Greedy algorithm (Charikar 2000). Iteration *i*, with $2 \le i \le k$, first computes the set of covered nodes in order to find those nodes that have to be removed (or retained). For this purpose, we sort the nodes in $C_{j-1}$ based on their weighted degree in $O(n \log n)$ time. Thus the overall time complexity of IWDS is $O(k m n \log n)$.

## Experiments

In this section, we provide an experimental evaluation of IWDS on synthetic and real networks.^1^ The design of a strong evaluation scheme for our algorithm is not simple, since we have to face two main issues:

1. Existing methods for computing the top *k* overlapping subgraphs (Galbrun et al. 2016) are defined for unweighted graphs and cannot be used on dual networks.
2. Existing network alignment algorithms do not aim to extract top *k* densest subgraphs.

Consequently, we cannot easily compare our approach with the existing state of the art methods, and we design an ad-hoc procedure for the evaluation of our method based on the following steps. First, we consider the performance of our approach on synthetic networks. In this way, we show that, in many of the cases we considered, IWDS can correctly recover top *k* weighted densest subgraphs. Then we apply our method to four real-world dual networks.

The alignment algorithm described of “A heuristic for Weighted-top-k-overlapping DCS” section is implemented in Python 3.7 using the NetworkX package for managing networks (Hagberg et al. 2008). IWDS is implemented in MATLAB R2020a. We perform the experiments on MacBook-Pro (OS version 10.15.3) with processor 2.9 GHz Intel Core i5 and 8 GB 2133 MHz LPDDR3 of RAM, Intel Iris Graphics 550 1536 MB.

### Synthetic networks

In the first part of our experimental evaluation, we analyse the performance of IWDS to find planted ground-truth subgraphs on synthetic datasets.

**Datasets.** We generate two noiseless synthetic datasets, consisting of $k=5$ planted dense subgraphs (cliques). *Synthetic1* contains five non-overlapping ground-truth subgraphs, while *Synthetic3* contains five overlapping ground-truth subgraphs.

In *Synthetic1*, each planted dense subgraph contains 30 nodes and has edge weights randomly generated in the interval [0.8, 1]. In *Synthetic3*, each planted dense subgraph contains 20 nodes not shared with other planted subgraphs. The subgraphs are arranged in a cycle, 5 nodes of each subgraph are shared with the subgraph on one side and 5 nodes are shared with the subgraph on the other side. Edge weights are randomly generated in the interval [0.8, 1].

These cliques are then connected to a background subgraph of 100 nodes. We consider three different ways to generate the background subgraph: Erdös–Renyi with parameter $p=0.1$, Erdös–Renyi with parameter $p=0.2$ and Barabasi–Albert with parameter equal to 10. Weights of the background graphs are randomly generated in interval [0, 0.5]. Then 50 edges connecting cliques and the background graph are randomly added (with weights randomly generated in interval [0, 0.5]).

Based on this approach, we generate four different sets of synthetic networks, called *Synthetic1*, *Synthetic2*, *Synthetic3* and *Synthetic4*. *Synthetic1* (for the non-overlapping case) and *Synthetic3* (for the overlapping case) are generated as described above. *Synthetic2* and *Synthetic4*, respectively, are obtained by applying noise to the synthetic networks in *Synthetic1*, *Synthetic3*, respectively. The noise is added by varying 5%, 10% and 15% of node relations of each network. A set of pairs of nodes are chosen randomly: if they belong to the same clique, the weight of the edge connecting the two nodes is changed to a random value in the interval [0, 0.5]; else an edge connecting the two nodes is (possibly) added (if not already in the network) and its weight is randomly assigned a value in the interval [0.8, 1].

**Outcome.** We present the results of our experimental evaluation, in particular, the average running time, density, distance and F1-score,^2^ varying the parameter $\alpha$. We recall that F1-score is the average mean of precision and recall, and, as in Galbrun et al. (2016) we consider this measure to evaluate the accuracy of our method to detect the ground-truth subgraphs. Following Yang and Leskovec (2012), we consider the number of shared nodes between each ground-truth subgraph and each detected subgraph, so that we are able to define the best-matching of ground-truth subgraphs and detected subgraphs. Then, we compute the *F*1[*t*/*d*] measure as the average F1-score of the best-matching ground-truth subgraph to each detected subgraph (*truth to detected*) and *F*1[*d*/*t*] measure as the average F1-score of the best-matching detected subgraph to each ground-truth subgraph (*detected to truth*). Notice that in most of the cases considered, the running time of IWDS increases with the increasing of $\alpha$. Also, generally, the solutions returned by IWDS for larger values of $\alpha$ are denser than for small values, while the solutions with small values of $\alpha$ have a higher value of distance (hence the subgraphs returned have a smaller overlapping).

Tables 1 and 3 report average results of running time (in minutes), density, distance and F1 scores for the two noiseless datasets. Table 1 shows the experimental results for the noiseless *Synthetic1* dataset, where ground-truth subgraphs are disjoint. In this case IWDS is able to detect the ground-truth subgraphs for all values of $\alpha$, averaged over 300 examples. Table 2 shows the experimental results for the noiseless *Synthetic3* dataset, where ground-truth subgraphs are overlapping. In this case the best performances are achieved for $\alpha =0.75$, where F1[t/d] = 0.745, while F1[d/t]= 0.804. The experimental results show that F1[d/t] increases with $\alpha$, in particular for lower values of $\alpha$ ($\alpha \le 0.25$) the performance of IWDS for this measure is poor. We observe that for values of $\alpha \ge 0.5$, the F1[t/d] measure decreases as $\alpha$ increases.

**Table 1:** Performance of IWDS on non overlapping generated networks (called *synthetic1*) for $k=5$, varying $\alpha$ from 0.05 to 0.9, the running time (in minutes), the density and the distance are averaged over 300 examples

	$\alpha =0.05$	$\alpha =0.1$	$\alpha =0.25$	$\alpha =0.5$	$\alpha =0.75$	$\alpha =0.9$
Time	0.0188	0.0187	0.0194	0.0217	0.0259	0.0231
Density	65.28	65.28	65.28	65.28	65.28	65.28
Distance	20	20	20	20	20	20
*F*1[*t*/*d*]	1.00	1.00	1.00	1.00	1.00	1.00
*F*1[*d*/*t*]	1.00	1.00	1.00	1.00	1.00	1.00

**Table 2:** Performance of IWDS on overlapping generated networks (called *synthetic3*) for $k=5$, varying $\alpha$ from 0.05 to 0.9, the running time (in minutes), the density and the distance are averaged over 300 examples

	$\alpha =0.05$	$\alpha =0.1$	$\alpha =0.25$	$\alpha =0.5$	$\alpha =0.75$	$\alpha =0.9$
Time	0.0104	0.0128	0.0145	0.0170	0.0188	0.0209
Density	21.00	23.41	32.29	46.11	57.95	64.22
Distance	18.178	17.473	16.321	15.853	15.741	15.344
*F*1[*t*/*d*]	0.509	0.415	0.689	0.768	0.745	0.456
*F*1[*d*/*t*]	0.101	0.157	0.331	0.583	0.804	0.923

Tables 3 and 4 show the performances of IWDS on the noisy datasets *Synthetic2* and *Synthetic4*. Recall that for these datasets, we consider noise values of 0.05, 0.10 and 0.15. The results we present are averaged over 90 examples. As for the noiseless datasets, we vary the value of parameter $\alpha$.

**Table 3:** Performance of IWDS on non overlapping generated networks with added noise varying from 0.05 to 0.15 (called *synthetic2*) for $k=5$, varying $\alpha$ from 0.05 to 0.9, the running time (in minutes), the density and the distance are averaged over 90 examples

Noise	$\alpha =0.05$	$\alpha =0.1$	$\alpha =0.25$	$\alpha =0.5$	$\alpha =0.75$	$\alpha =0.9$
0.05						
Time	0.0181	0.0181	0.0203	0.0214	0.0222	0.0236
Density	65.46	65.46	65.48	65.53	65.55	65.55
Distance	20	19.998	19.996	19.996	19.991	19.850
*F*1[*t*/*d*]	0.989	1.00	1.00	1.00	1.00	1.00
*F*1[*d*/*t*]	0.990	0.991	0.993	0.996	0.998	0.995
0.10						
Time	0.0187	0.0179	0.0207	0.0199	0.0233	0.0238
Density	65.42	65.42	65.53	65.72	65.89	66.00
Distance	19.999	19.999	19.986	19.976	19.960	19.847
*F*1[*t*/*d*]	0.978	0.968	1.00	1.00	1.00	1.00
*F*1[*d*/*t*]	0.960	0.962	0.970	0.982	0.992	0.994
0.15						
Time	0.0126	0.0131	0.0164	0.0194	0.0230	0.0241
Density	36.63	39.35	43.03	51.57	59.67	64.47
Distance	19.439	19.111	18.218	18.112	18.083	18.056
*F*1[*t*/*d*]	0.93	0.95	0.93	0.98	0.95	0.94
*F*1[*d*/*t*]	0.41	0.47	0.54	0.70	0.85	0.94

**Table 4:** Performance of IWDS on overlapping generated networks with added noise varying from 0.05 to 0.15 (called *synthetic4*) for $k=5$, varying $\alpha$ from 0.05 to 0.9, the running time (in minutes), the density and the distance are averaged over 90 examples

Noise	$\alpha =0.05$	$\alpha =0.1$	$\alpha =0.25$	$\alpha =0.5$	$\alpha =0.75$	$\alpha =0.9$
0.05						
Time	0.0090	0.0112	0.0149	0.0180	0.0203	0.0205
Density	21.34	24.89	32.09	45.92	57.31	63.50
Distance	18.361	17.506	15.823	15.550	15.220	15.024
*F*1[*t*/*d*]	0.649	0.660	0.563	0.692	0.631	0.527
*F*1[*d*/*t*]	0.131	0.228	0.332	0.589	0.806	0.927
0.10						
Time	0.0098	0.0118	0.0137	0.0178	0.0195	0.0212
Density	21.95	25.54	32.72	46.35	58.43	65.25
Distance	18.275	17.260	15.761	15.229	14.876	13.847
*F*1[*t*/*d*]	0.648	0.568	0.567	0.595	0.548	0.463
*F*1[*d*/*t*]	0.131	0.225	0.330	0.581	0.807	0.936
0.15						
Time	0.0092	0.0113	0.0149	0.0178	0.02	0.0218
Density	22.40	26.06	32.98	46.68	58.91	65.75
Distance	18.213	17.189	15.332	14.932	14.263	12.717
*F*1[*t*/*d*]	0.624	0.555	0.501	0.539	0.419	0.303
*F*1[*d*/*t*]	0.134	0.223	0.336	0.586	0.811	0.938

For *Synthetic2*, for noise value 0.05 and 0.10, we obtain near optimal solutions for all the cases considered. The performances of IWDS starts to degrade with noise equal to 0.15, in particular the values of F1[d/t] for $\alpha \le 0.25$. F1[t/d] is instead close to 1 (at least 0.93) for the values of $\alpha$ considered.

For *Synthetic4*, the added noise has a significant impact on the quality of computed solutions, even for noise value equal to 0.05. While the noise increasing has a limited effect on IWDS for small value of $\alpha$ ($\alpha \le 0.25$), for higher values of $\alpha$ leads to a degrade in performance, in particular for F1[t/d].

### Dual networks

We evaluate IWDS on four real-world dual network datasets:

**Datasets.** *G-graphA*. The G-graphA dataset is derived from the GoWalla social network where users share their locations (expressed as GPS coordinates) by checking-in into the web site (Cho et al. 2011). Each node represents a user and each edge links two friends in the network. We obtained the physical network by considering friendship relation on the social network. We calculated the conceptual network by considering the distance among users. Then we run the first step of our algorithm and we obtained the alignment graph *G-graphA*, containing 2,241,339 interactions and 9878 nodes (we set $\delta$=4). In this case a DCS represents set of friends that share check-ins in near locations.

*DBLP-graphA*. The *DBLP-graphA* dataset is extracted from a computer science bibliography and represents interactions between authors. Nodes represent authors and edges represent connections between two authors if they have published at least one paper together. Each edge in the physical network connects two authors that co-authored at least one paper. Edges in the conceptual network represent the similarity of research interests of the authors calculated on the basis of all their publications. After running the first step of the algorithm (using $\delta$=4), we obtained an alignment graph *DBLP-graphA* dataset containing 553,699 interactions and 18,954 nodes. In this case a DCS represents a set of co-authors that share some strong common research interests and the use of DNs is mandatory, since physical network shows only co-authors that may not have many common interests and the conceptual network represents authors with common interest that may not be co-authors.

*HS-graphA*. *HS-graphA* is a biological dataset and is taken from the STRING database (Szklarczyk et al. 2016). Each node represents a protein, and each edge takes into account the reliability of the interactions. We use two networks for modelling the database: a conceptual network represents such reliability value; and a physical network stores the binary interactions. The *HS-graphA* dataset contains 5,879,727 interactions and 19,354 nodes (we set $\delta$=4).

*Protein-interaction* We extracted from the STRING database a subnetwork of proteins involved into the SARS-COV-2 infection (Szklarczyk et al. 2016). The physical network contains interacting proteins, while the conceptual network contains the strength of the association among them. *Protein-Interaction* contains 192 nodes and 418 edges (Table 5).

**Table 5:** Properties of the alignment graphs obtained for each dataset

Graph	Represented relation	Nodes	Edges
DBLP-graphA	Co-authorship	18,954	553,699
G-graphA	Social	9878	2,241,339
HS-graphA	Protein interactions	19,354	5,879,727
Protein-interaction	Protein interactions	192	418

**Outcome**

For these large size datasets, we set the value of *k* to 20, following the approach in Galbrun et al. (2016). Table 6 reports the running time of IWDS, and the density and distance of the solutions returned by IWDS. As for the synthetic datasets, we consider six different values of $\alpha$. As shown in Table 6, by increasing the value of $\alpha$ from 0.05 to 0.5, IWDS (except of one case, *HS-graphA* with $\alpha =0.1$) returns solutions that are denser, but with lower distance.

Table 6 shows also how the running time of IWDS is influenced by the size of the network and by the value of $\alpha$. We have put a bound of 20 h on the running time of IWDS and the method was not able to return a solution for HS-graphA for $\alpha \ge 0.5$ within this time. The running time is influenced in particular by the number of edges of the input network. DBLP-graphA and HS-graph-A have almost the same number of nodes, but HS-graph-A is much more denser than DBLP-graphA. IWDS for the former network is remarkably slower than for DBLP-graphA (1.986 slower for $\alpha =0.05$, 6.218 slower for $\alpha =0.25$). The running time of IWDS is considerably influenced by the value of parameter $\alpha$, since it increases as $\alpha$ increases. Indeed by increasing the value of $\alpha$, less nodes are removed by Case 1 and Case 2 of IWDS, hence in iterations of IWDS V-Greedy is applied to larger subgraphs. This fact can be seen in particular for HS-graphA, for which IWDS failed to terminate within 20 h when $\alpha \ge 0.5$.

**Table 6:** Performance of IWDS on real-world network for $k=20$, varying $\alpha$ from 0.05 to 0.9. For each network, we report the running time in minutes, the density and the distance

Set	$\alpha =0.05$	$\alpha =0.1$	$\alpha =0.25$	$\alpha =0.5$	$\alpha =0.75$	$\alpha =0.9$
Alignment-graph						
Time	0.055	0.058	0.062	0.065	0.068	0.068
Density	28.14	30.45	37.14	46.44	47.73	52.94
Distance	378.76	373.61	359.94	351.50	347.81	339.17
G-graphA						
Time	89.84	98.72	184.87	336.72	426.56	486.68
Density	2863.99	4000.73	6345.67	10989.07	9297.13	10737.01
Distance	275.82	257.84	220.16	210.79	196.06	193.02
DBLP-graphA						
Time	105.69	125.71	165.25	212.07	251.08	277.39
Density	39.61	52.39	74.12	91.13	97.25	98.78
Distance	307.72	231.25	213.04	204.37	198.54	196.96
HS-graphA						
Time	209.88	749.06	1027.58	–	–	–
Density	1326.07	1153.68	1799.22			
Distance	226.40	212.34	205.55			

### Biological evaluation of results

For biological data there is the possibility to evaluate the relevance of the results considering the relevance of the biological knowledge that results may convey.

Biological data are usually annotated with terms extracted from ontologies, e.g. Gene Ontology (Guzzi et al. 2012). Consequently, experiments of analysis of biological data may evaluated in terms of the biological knowledge inferred from the analysis of data and in terms of the statistical relevance of the results themselves. For instance, given a DCS extracted from two biological networks, it is interesting to determine the biological meaning of the DCS and how this is relevant, i.e. how this DCS may convey biological relevance with respect to another random one. Usually, subgraphs of biological networks may represent groups of interacting proteins sharing some common functions or playing similar biological roles. Consequently, it is possible to evaluate the biological relevance of obtained results by considering the role of proteins. Such information are stored and organised into biological ontologies such as Gene Ontology (GO) (Harris et al. 2004). GO functional enrichment has been proposed to evaluate the significant presence of common roles or function in a solution represented as a list of genes/proteins. It has been shown that the use of semantic similarities (SS) (Guzzi et al. 2012) is a feasible and efficient way to quantify biological similarity among proteins. SS measures are able to quantify the functional similarity of pairs of proteins/genes, comparing the GO terms that annotate them, therefore proteins that share the biological role have high values of semantic similarity. As a consequence, genes/proteins that are found in the same solution should have a semantic similarity significantly higher than random expectation. These considerations have been used during the design of the evaluation of our results that we adapted from the evaluation scheme proposed in Mina and Guzzi (2014).

Given a DCS $DCS_k$ we calculate its internal semantic similarity $SS_{DCS_k}$ as the average semantic similarity of all the nodes pairs of the DCS as follows:

1 $$\begin{aligned} SS_{DCS_k} =\frac{\sum _{n_i \in DCS_k}\sum _{n_j \in DCS_k,j \ne i} SS_{DCS_k}(n_i,n_j)}{\Vert SS_{DCS_k}\Vert \Vert SS_{DCS_k-1}\Vert } \end{aligned}$$

We compare the DCS extracted from the biological network against random ones obtained by randomly sampling the input networks to prove their statistical significance. Given a DCS $DCS_i$, we can test the null hypothesis: $H_1^0$: the average semantic similarity of the protein internals to the DCS $SS(DCS_i)$ is higher than by chance, where the background distribution can be estimated from the semantic similarity of random subgraphs $RS_i$ taken from the alignment graph $SS(RS_i)$, using for instance 0.05 as significance level.

Consequently we design this test as described in the following algorithm:

- Let $DCS_{i}$ be a given DCS;
- Let $SS(DCS_{i})$ be its internal semantic similarity
- Let $V_s$ be the set of 100 random subgraph with same size $V_s$={$RS_{j}$} j=0,..,99
- For Each $RS_{j} \in V_s$ calculate $SS_j(RS_{j})$ the internal semantic similarity of each random solution
- Compare $SS(DCS_{i})$ and all the $SS_j(RS_{j})$ using a non parametric test
- Accept or Refuse the Hypothesis $SS(DCS_{i})$ is significantly higher than $SS_j(RS_{j})$

Consequently, for each graph in the solution we generate 100 random graphs of the same size, by sampling the obtained alignment graph. For each graph we calculated its internal semantic similarity using the Resnick measure (Resnik 1999). Results demonstrate that our solution is biologically relevant and the relevance is higher than by chance as summarised in Table 7.

**Table 7:** Comparison of the average semantic similarity for the two biological networks considered

	Semantic similarity
Random solutions	$0.3 \pm 0.1$
DCS	$0.6 \pm 0.1$

## Conclusion

DNs are used to model two kinds of relationships among elements in the same scenario. A DN is a pair of networks that have the same set of nodes. One network has unweighted edges (physical network), while the second one has weighted edges (conceptual network). In this contribution, we introduced an approach that first integrates a physical and a conceptual network into an alignment graph. Then, we applied the Weighted-Top-k-Overlapping DCS problem to the alignment graph to find *k* dense connected subgraphs. These subgraphs represent subsets of nodes that are strongly related in the conceptual network and that are connected in the physical one. We presented a heuristic, called IWDS, for Weighted-Top-k-Overlapping DCS and an experimental evaluation of IWDS. We first considered as a proof-of-concept the ability of our algorithm to retrieve known densest subgraphs in synthetic networks. Then we tested the approach on four real networks to demonstrate the effectiveness of our approach. Future work will consider a possible high performance implementation of our approach and the application of the IWDS algorithm to other scenarios (e.g. financial or marketing datasets).

## Data Availability

https://github.com/mehdihosseinzadeh/-k-overlapping-densest-connected-subgraphs

## Abbreviations

DN: (Dual networks)
DCS: (Densest connected subgraph)
LNA: (Local network alignment)
IWDS: (Iterative weighted dense subgraphs)

## Footnotes

1 The source code and data used in our experiments are available at https://github.com/mehdihosseinzadeh/-k-overlapping-densest-connected-subgraphs.

2 Given the ground-truth and detected subgraphs, F1-score is calculated considering precision and recall, where precision is the fraction of the number of nodes in the ground-truth correctly identified by detected subgraphs divided by number of nodes in the detected subgraphs, whereas the recall is the fraction of the number nodes in the ground-truth correctly identified by the detected subgraphs divided by the number of nodes in the ground-truth.
